# Adenosine administration in supraventricular tachycardia

**DOI:** 10.1007/s12471-017-1032-x

**Published:** 2017-09-12

**Authors:** P. Robles Velasco, I. Monedero Sánchez, A. Rubio Caballero, M. Chichakli Cela, Y. González Doforno

**Affiliations:** Cardiology Unit, Hospital Universitario Fundación Alcorcon, Madrid, Spain

A 58-year-old male patient, active smoker, had been previously assessed for paroxysmal palpitations, with the following baseline electrocardiogram (ECG) (Fig. 1). Structural heart disease had been excluded by echocardiography. He was admitted to the emergency room with these symptoms, and the ECG then showed irregular narrow complex tachycardia (Fig. 2). Although F waves were visible in the inferior leads, the patient was given 6 mg of intravenous adenosine by the emergency physicians to better determine the underlying tachycardia and make a differential diagnosis with other supraventricular tachycardias. Was this the correct approach?

**Fig. 1:**
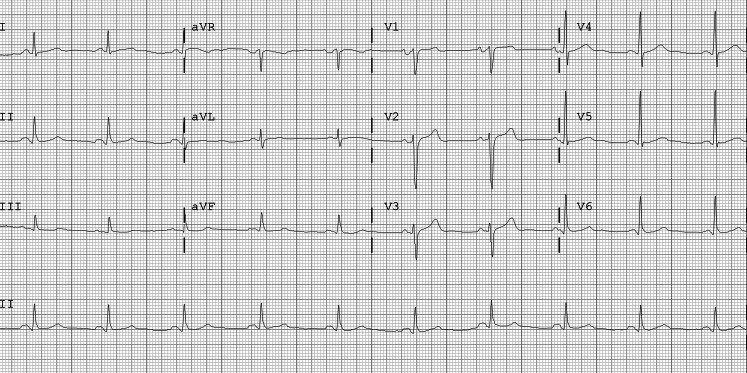
Baseline ECG showed sinus rhythm and no significant alterations

**Fig. 2:**
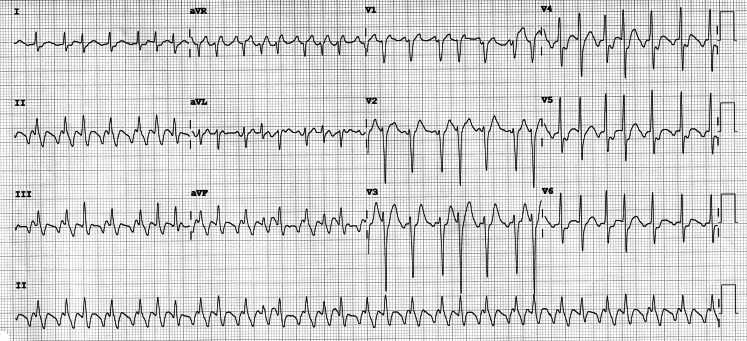
ECG during symptoms showed irregular narrow complex tachycardia, with F waves visible in the inferior leads

## Answer

You will find the answer elsewhere in this issue.

